# A deep learning approach for dental implant planning in cone-beam computed tomography images

**DOI:** 10.1186/s12880-021-00618-z

**Published:** 2021-05-19

**Authors:** Sevda Kurt Bayrakdar, Kaan Orhan, Ibrahim Sevki Bayrakdar, Elif Bilgir, Matvey Ezhov, Maxim Gusarev, Eugene Shumilov

**Affiliations:** 1 Department of Periodontology, Faculty of Dentistry, Eskisehir Osmangazi University, Eskişehir, Turkey; 2 Department of Oral and Maxillofacial Radiology, Faculty of Dentistry, Ankara University, 06500 Ankara, Turkey; 3 Medical Design Application and Research Center (MEDITAM), Ankara University, Ankara, Turkey; 4 Department of Oral and Maxillofacial Radiology, Faculty of Dentistry, Eskisehir Osmangazi University, Eskişehir, Turkey; 5 Eskisehir Osmangazi University Center of Research and Application for Computer Aided Diagnosis and Treatment in Health, Eskisehir, Turkey; 6 Diagnocat, Inc, San Francisco, USA

**Keywords:** Artificial intelligence, Dental implant, Implant planning, Dentistry

## Abstract

**Background:**

The aim of this study was to evaluate the success of the artificial intelligence (AI) system in implant planning using three-dimensional cone-beam computed tomography (CBCT) images.

**Methods:**

Seventy-five CBCT images were included in this study. In these images, bone height and thickness in 508 regions where implants were required were measured by a human observer with manual assessment method using InvivoDental 6.0 (Anatomage Inc. San Jose, CA, USA). Also, canals/sinuses/fossae associated with alveolar bones and missing tooth regions were detected. Following, all evaluations were repeated using the deep convolutional neural network (Diagnocat, Inc., San Francisco, USA) The jaws were separated as mandible/maxilla and each jaw was grouped as anterior/premolar/molar teeth region. The data obtained from manual assessment and AI methods were compared using Bland–Altman analysis and Wilcoxon signed rank test.

**Results:**

In the bone height measurements, there were no statistically significant differences between AI and manual measurements in the premolar region of mandible and the premolar and molar regions of the maxilla (*p* > 0.05). In the bone thickness measurements, there were statistically significant differences between AI and manual measurements in all regions of maxilla and mandible (*p* < 0.001). Also, the percentage of right detection was 72.2% for canals, 66.4% for sinuses/fossae and 95.3% for missing tooth regions.

**Conclusions:**

Development of AI systems and their using in future for implant planning will both facilitate the work of physicians and will be a support mechanism in implantology practice to physicians.

## Background

Dental implants have been preferred by clinicians for many years in cases of the total, partial and single-tooth edentulism [1–3]. Detailed planning before the implant operation increases the success of the treatment due to the facility of placing in the correct position of the implant and eliminating the surgical risks [4–6]. For this purpose, in implant surgery, the various radiographic techniques are used to evaluate alveolar bone features (bone quality, thickness, and height) and anatomical variations in the operation area (such as nasal fossa, mandibular canal, mental foramen and sinuses) [4, 5, 7].

Panoramic and intraoral radiographs are used still in dental implant practices to provide an overview of the jaws and to create a preliminary idea; but these radiographic techniques are insufficient for detailed implant planning [4, 8, 9]. Cross-sectional tomograms such as computed tomography (CT) and cone-beam computed tomography (CBCT) which offer three-dimensional (3D) information to surgeons are currently used as an alternative to these conventional techniques [9]. CBCT devices developed for dentomaxillofacial imaging, have more affordable prices and smaller device sizes than CT devices. It also offers high-quality images at a lower radiation dose and short scanning time [4, 9, 10]. It is known that CBCT devices are very successful in determining the ideal implant sizes (i.e., length and width) before the operation and in predicting the necessary extra surgical procedures (i.e., guided tissue regeneration, splitting, sinus elevation) in case of insufficient bone in the operation site [4, 5, 7, 11]. Nevertheless, the physician's knowledge, skills, and experience in the interpretation of CBCT images also play very great roles in performing detailed implant planning [12].

Artificial intelligence (AI) is a field of computer science aimed at performing various specific functions that require human intelligence. It imitates human intelligence and improves its these features acquired over time using the deep learning methods [13]. In radiological diagnostic clinics, using the AI has provided to emerge the computer-aided diagnosis (CAD) systems. Then the development of this system has gained momentum in many fields of medicine and its use also has become widespread in health sectors such as dentistry in recent years [14, 15]. A deep convolutional neural network method (DCNN) is a powerful deep learning application used on medical diagnostic images [16, 17]**.** There are studies in the literature where this method, which also enables the processing of more complex images such as CBCT images, has used in various diagnostics in dentistry such as tooth numbering, periapical pathosis, and mandibular canal detection [18–21].

Many specialists and general practitioners have not received extensive training on radiographic image evaluation and not competent in detailed implant planning and interpretation of anatomical data [10, 22]. This is a situation that makes challenges of dentistry practice and still awaits a solution. Using AI systems in radiographic interpretation provides many advantages to the physician and can contribute to solving this problem. Also, it may prevent wrong diagnosis and treatment planning (which may be due to work intensity, carelessness or inexperience), unnecessary loss of time/workload in dentistry [12].

To our best knowledge, there are no studies in the literature where AI systems are used in implant planning. The purpose of this study was to verify the diagnostic performance and assess the reliability of an artificial intelligence system based on the deep convolutional neural network method to implant planning in CBCT images.

## Methods

### Patient selection and imaging

A total of 75 patients’ (cases with implant indication and recorded in 2019) CBCT images obtained from the CBCT archive of the Faculty of Dentistry of Eskişehir Osmangazi University were included in the study. Also, 508 measurements (in areas that have missing teeth and with implant indication) were performed from them. For the study procedures, the Non-interventional Clinical Research Ethics Committee Approval was received and principles of the Declaration of Helsinki were followed at each stage (decision date and number: 08.07.2019 and 2019-220). All images were acquired with the same CBCT scanner (Promax 3D Mid; Planmeca, Helsinki, Finland) and the same conditions. Diagnostic settings were as follows: 94 kVp, 14 mA, 360° rotation, 27 s.

### Evaluation of tomography data

All images were examined by an oral and maxillofacial radiologist with at least 8 years of professional experience (İ.Ş.B) by converting them to DICOM format. The jaws were separated as mandible/maxilla and each jaw was grouped as anterior/premolar/molar teeth region. Canine teeth and incisors were included in the anterior region. Canals/sinuses/fossae associated with alveolar bones were detected and missing teeth were recorded. In missing teeth areas, bone height and thickness were measured by manual assessment methods using InvivoDental 6.0 (Anatomage Inc. San Jose, CA, USA). In other words, all evaluations were performed as in implant plannings; and limitations of anatomical structures were taken into consideration.

After manual evaluations, all files were randomly uploaded to the deep convolutional neural network (Diagnocat, Inc.) for determinations of canals/sinuses/fossae and calculation of bone length/width in missing teeth areas. The data obtained from manual assessment and artificial intelligence (AI) methods were compared.

### Model pipeline

Diagnocat AI system prepares an implant planning report based on a pipeline of multiple pre-trained fully convolutional networks and algorithmic slice extraction. Predictions crucial for implant planning include voxel-perfect segmentations of teeth, jaws, mandibular canals, maxillary sinuses, and missing teeth.

**Fig. 1:**
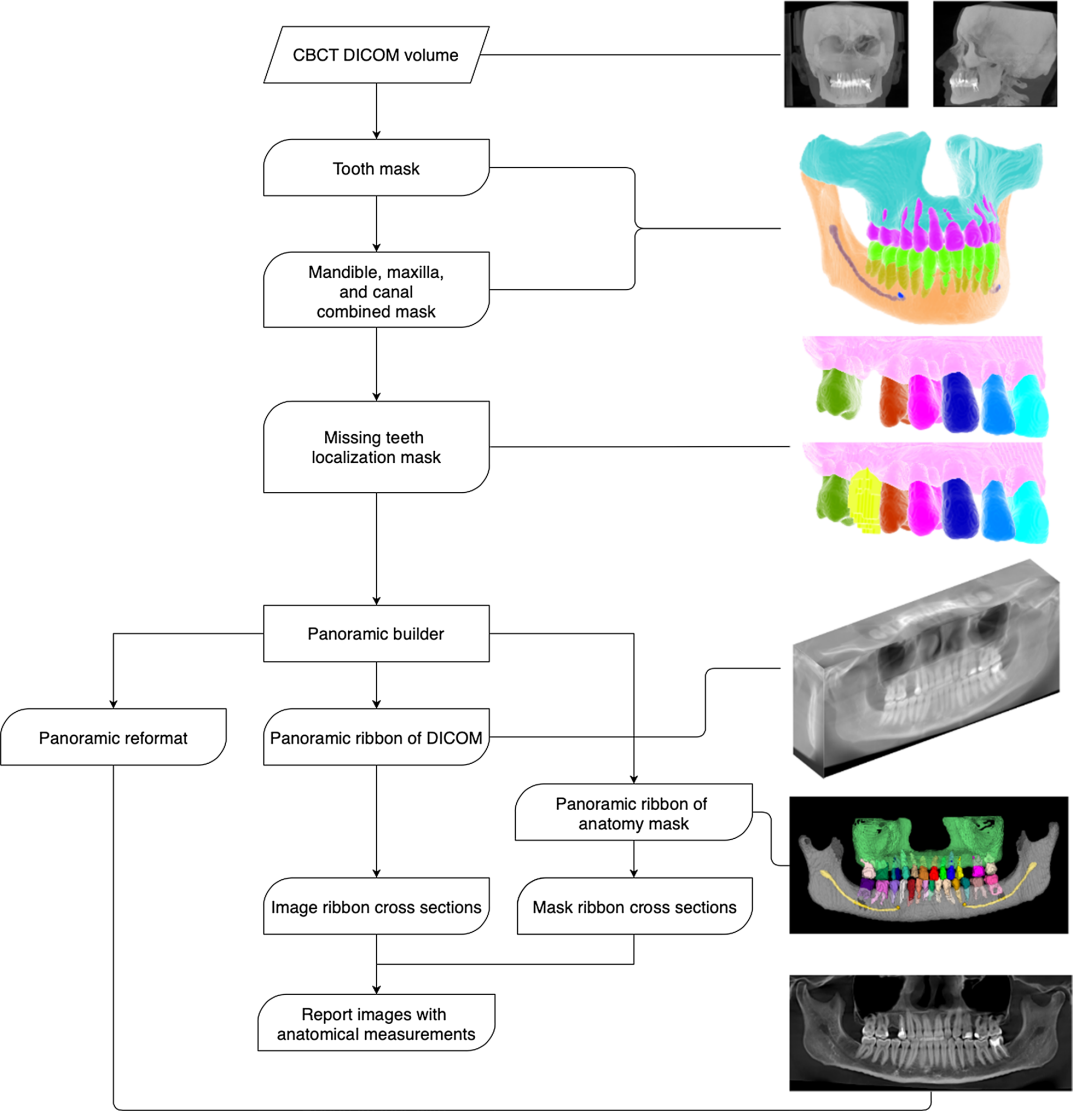
AI model pipeline to implant planning procedure

Missing teeth segmentation relies on both present teeth and jaws segmentations. A missing tooth mask is a virtual tooth mask that is extracted using neighboring teeth location, tilt, and placement according to a jaw. It allows to predict a mesiodistal angle of implant placement and provides a guide to a slicing algorithm. Implant planning study includes a panoramic reformat of a specified jaw and a slice section with vestibulooral slice orientation. Teeth, jaws, and mandibular canal segmentations are used to build a panoramic ribbon of both a study image and a combined segmentation mask. All slices in a study are extracted from a region of interest (RoI) of a panoramic image ribbon with a user chosen step, 2 mm by default, and slice thickness, 1 voxel by default. Predicted target (missing) tooth and neighboring (potentially missing) teeth masks define a RoI and mesiodistal angle of slice extraction showing possible implant placement (Fig. 1).

**Fig. 2:**
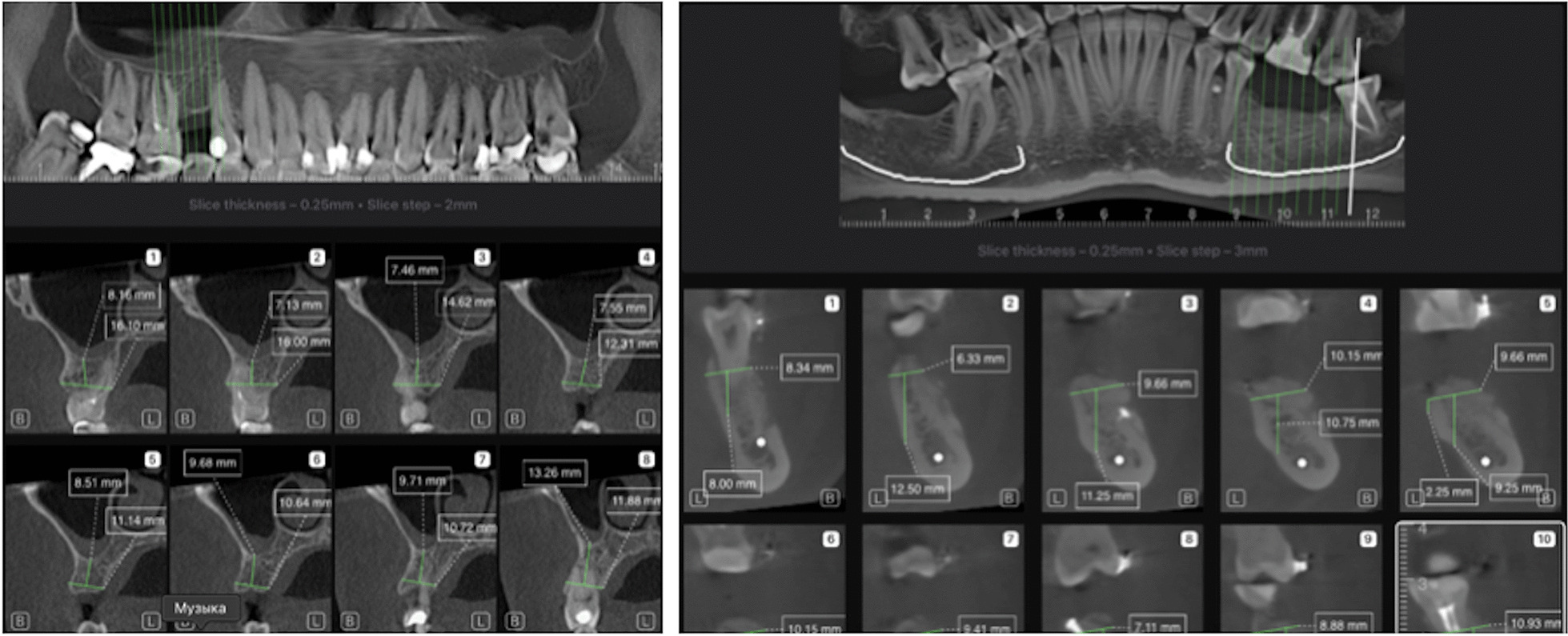
Implant planning report which was created otomatically for maxilla and mandibula case using the AI

Every slice has two to three measurements providing information about possible implant size and direction. The first measurement shows a width of alveolar bone. The second measurement shows a distance from the first measurement line to the closest obstacle in implant direction which is either of mandibular canal, maxillary sinus, or a jaw bone edge. The third measurement is calculated only in mandible case and shows a vertical distance from an oral end of the first measurement line to a mandible bone edge (Fig. 2).

### The architecture of the deep convolutional neural networks

Diagnocat AI system exploits a set of pre-trained semantic segmentation networks based on internally modified fully convolutional 3D U-Net architecture from [23] to obtain voxel-perfect segmentation masks of present teeth and anatomy elements and to approximate localization of missing teeth.

### Examiner consistency

The measurements were performed by the same examiner. One hundred measurements were repeated 1 week after the first evaluation by the examiner. In this way, intra-examiner agreement and reliability were evaluated using intraclass correlation coefficients and intra-evaluator technical error measurement (TEM) calculations. The intraclass correlation coefficient (95% confidence interval) was 0.995 (0.992–0.997) for bone thickness; 0.996 (0.994–0.997) for bone height. Also, relative TEM was 3.14 (acceptable) and reliability was 99.5% for bone thickness measurement, relative TEM was 2.37 (acceptable) and reliability was 99.6% for bone height measurements.

#### Statistical analyses

Statistical analyses were performed with the SPSS 21.0 Package Data Program (SPSS 21.0 Software Package Program, Inc., Chicago, IL). Kolmogorov–Smirnov test was used when testing for normality. A comparison of measurements calculated by manual assessment and artificial intelligence (AI) was made by Wilcoxon signed‐rank test and Bland–Altman analysis. A value of *p* < 0.05 was considered statistically.

## Results

Correctness frequencies of canal/sinus/fossa/missing tooth detections of the AI system are listed in Table 1. The percentage of right detection was 72.2% for canals, 66.4% for sinuses/fossae. Also, it was seen that 484 (95.3%) of 508 missing tooth regions were detected correctly and only 24 (4.7%) was detected incorrectly.

**Table 1:** False and right percentages of canal/sinus/fossa detections of the AI system

Regions	Canal/sinus/fossa detection (%, n)				Missing tooth detection (%, n)			
	Canal detection (Mandibula)		Sinus/fossa detection (Maxilla)		Mandibula		Maxilla	
	False	Right	False	Right	False	Right	False	Right
Anterior	83% (n = 44)	17% (n = 9)	91.8% (n = 56)	8.2% (n = 5)	9.4% (n = 5)	90.6% (n = 48)	8.2% (n = 5)	91.8% (n = 56)
Premolar	29.5% (n = 18)	70.5% (n = 43)	27.7% (n = 28)	72.3% (n = 73)	3.3% (n = 2)	96.7% (n = 59)	5.9% (n = 6)	94.1% (n = 95)
Molar	2.5% (n = 3)	97.5% (n = 117)	7.1% (n = 8)	92.9% (n = 104)	1.7% (n = 2)	98.3% (n = 118)	3.6% (n = 4)	96.4% (n = 108)
Total	27.8% (n = 65)	72.2% (n = 169)	33.6% (n = 92)	66.4% (n = 182)	3.8% (n = 9)	96.2% (n = 225)	5.5% (n = 15)	94.5% (n = 259)

The values of bone height and thickness measurements with the AI system and manual assessment are shown in Table 2. The AI system was unable to perform 80 of bone height measurements (therefore, bone height measurements evaluated on 428 images) and 15 of bone thickness measurements (therefore, bone thickness measurements evaluated on 493 images).

**Table 2:** Bone height and thickness measurements with the AI system and manual assessment

Regions		Parameters			
		Bone height measurements (n = 428)^#^		Bone thickness measurements (n = 493)^#^	
		AI	Manual	AI	Manual
		Median (min–max)	Median (min–max)	Median (min–max)	Median (min–max)
Mandibula	Anterior	19.2 (3.1–31.6)	12.8 (5.7–19.1)	5.7 (1.2–11.8)	4 (2.1–10.8)
	Premolar	**12 (1.2**–26.4)*	**12 (5**–19.9)*	6.1 (2.4–11.7)	4.6 (2.5–11.1)
	Molar	10 (1.2–19.2)	11,7 (2.6–20)	7.7 (4.5–13.8)	5.2 (3.3–12.5)
Maxilla	Anterior	24.3 (3.9–27.3)	13 (3.6—16,6)	6.5 (3.9–31.1)	4.4 (2.1–18.9)
	Premolar	**12.2 (2.3**–28.8)*	12.1 (2.4—22)*	7.2 (2–34.4)	4.9 (0–11.1)
	Molar	**6.9 (0.5**–26.3)*	**7.6 (0**–17.6)*	9.5 (1.6–29.5)	5.6 (0–15.2)

Bold variables in the table indicates no statistical significant difference (*p* > 0.05)

*There were no differences between AI and manual measurements for each parameter (*p* > 0.05). Min, minimum; Max, Maximum

^#^A total of 508 measurements were performed, but the AI system was unable to perform 80 of bone height measurements and 15 of bone thickness measurements. For each parameter, statistical analyzes were made on the measurements that can only be evaluated with the AI system

**Fig. 3:**
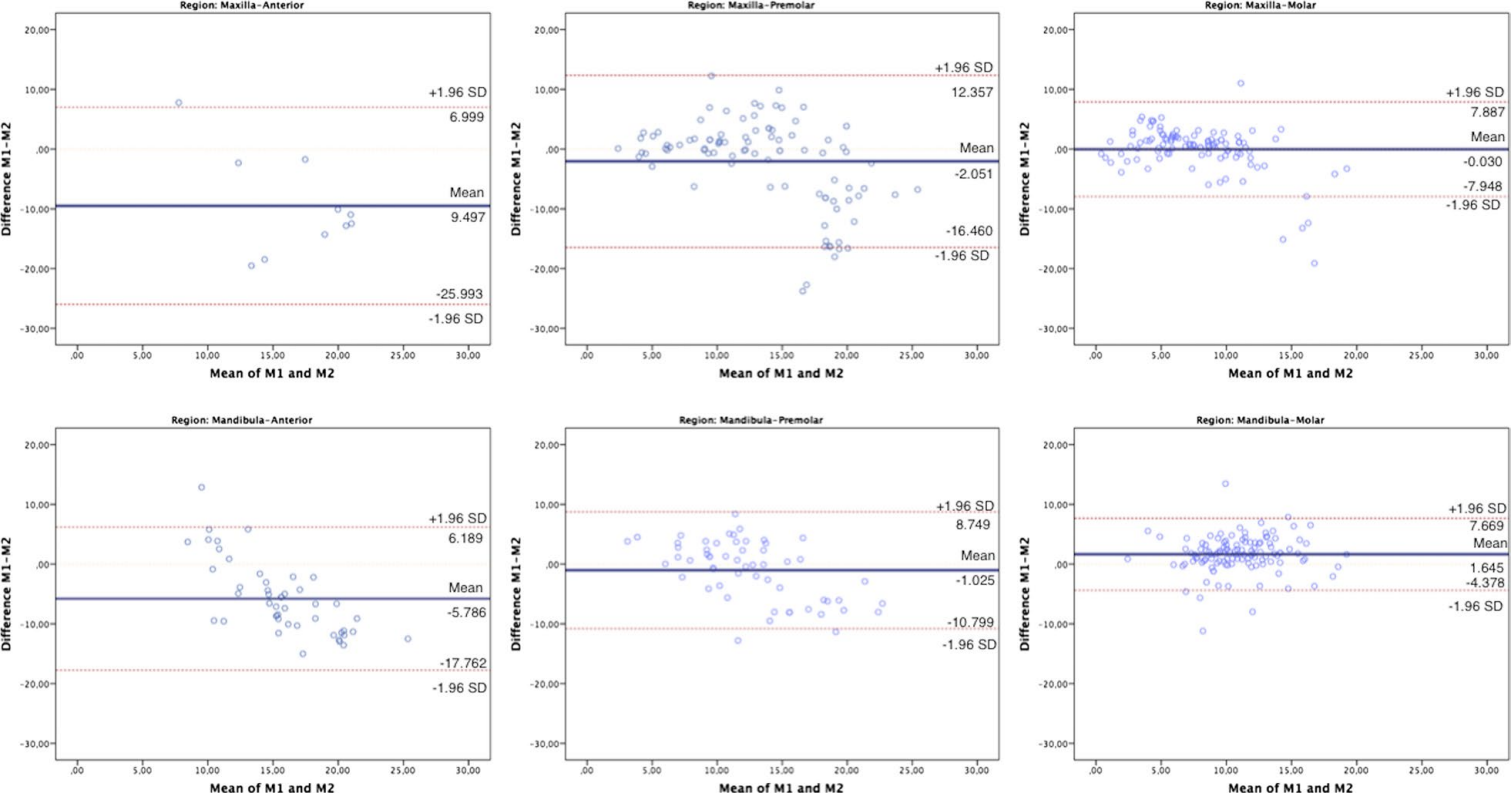
Bland Altman plots for bone height. M1, Manual measurements; M2, AI measurements

**Fig. 4:**
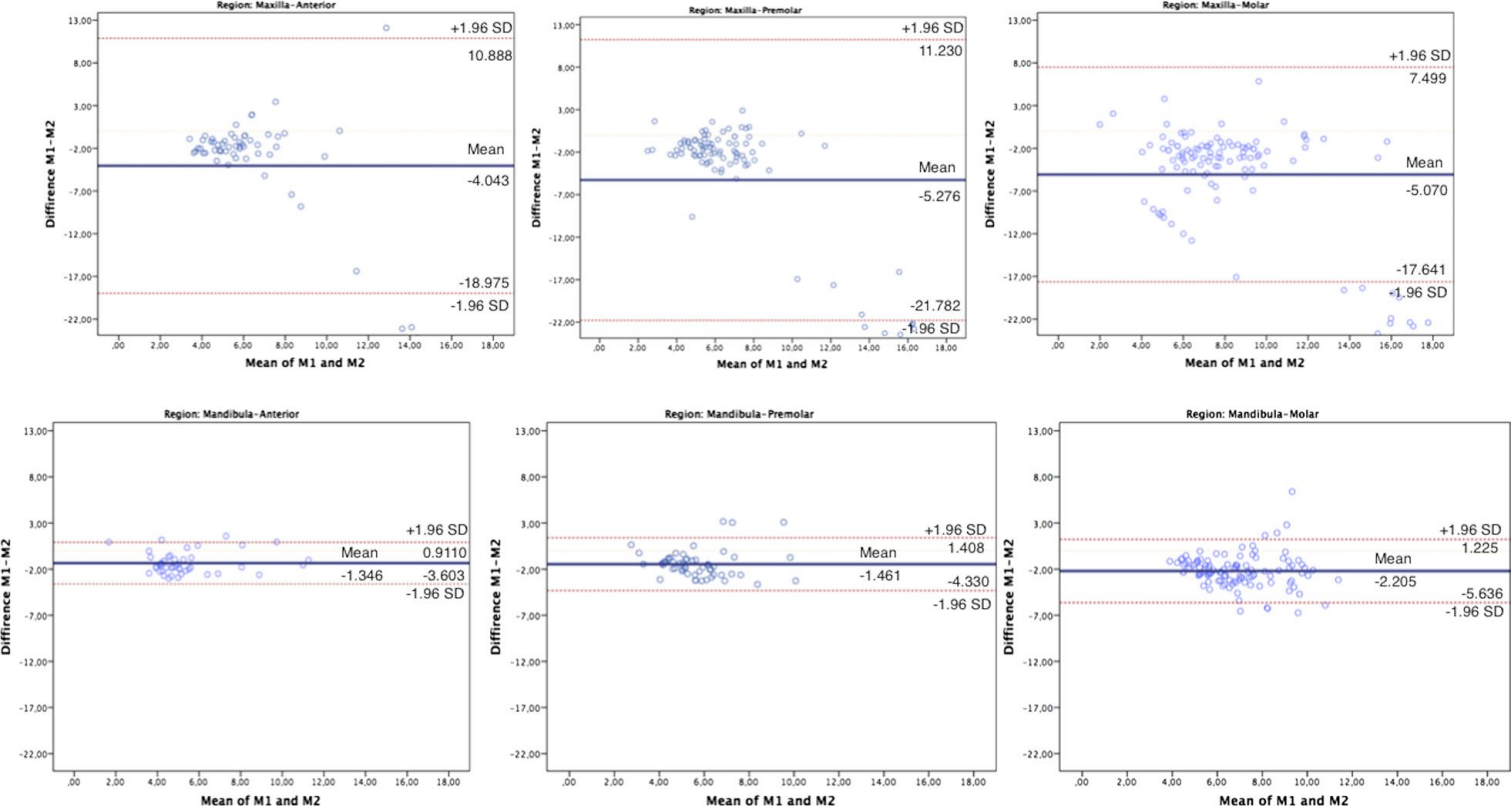
Bland Altman plots for bone thickness. M1, Manual measurements; M2, AI measurements

In the bone height measurements, there were no statistically significant differences between AI and manual measurements in the premolar region of mandible and the premolar and molar regions of the maxilla (*p* > 0.05). In the bone thickness measurements, there were statistically significant differences between AI and manual measurements in all regions of maxilla and mandible (*p* < 0.001). Bland Altman plots for measurements are shown in Figs. 3 and 4. Confidence interval for differences between the manual and AI system are shown in Table 3.

**Table 3:** Confidence interval for differences of manual and AI system

Parameters	Jaws	Regions	Paired differences					t	df	Sig (2-tailed)
			Mean	SD	Standard error mean	95% CI of the difference				
						Lower	Upper			
Bone height	Mandibula	Anterior	− 5.79	6.11	0.89	− 7.58	− 3.99	− 6.49	46	< 0.001
		Premolar	− 9.50	8.42	2.66	− 15.52	− 3.48	− 3.57	9	0,006
		Molar	− 1.02	4.99	0.66	− 2.35	0.30	− 1.55	56	0,126
	Maxilla	Anterior	− 2.05	7.35	0.77	− 3.58	− 0.52	− 2.66	90	0,009
		Premolar	1.64	3.07	0.28	1.08	2.20	5.81	117	< 0.001
		Molar	− 0.03	4.04	0.39	− 0.81	0.75	− 0.08	104	0,938
Bone thickness	Mandibula	Anterior	− 1.35	1.15	0.17	− 1.68	− 1.00	− 8.01	46	< 0.001
		Premolar	− 4.04	7.62	0.97	− 5.99	− 2.09	− 4.14	60	< 0.001
		Molar	− 1.46	1.46	0.19	− 1.85	− 1.07	− 7.53	56	< 0.001
	Maxilla	Anterior	− 5.28	8.42	0.84	− 6.95	− 3.60	− 6.26	99	< 0.001
		Premolar	− 2.20	1.75	0.16	− 2.52	− 1.89	− 13.68	117	< 0.001
		Molar	− 5.07	6.41	0.61	− 6.28	− 3.86	− 8.29	109	< 0.001

*CI* confidence interval

## Discussion

In recent years, there has been a significant increase in the number of studies using AI for purposes such as disease detection and classification, organ and lesion segmentation in the medicine [24, 25]. With all these developments, the use of AI systems to interpret radiological images in dental radiology has also become widespread [14, 15, 25]. Using computer-aided systems in imaging techniques that require experience and expertise such as especially CT and CBCT will provide great convenience to physicians. But new academical studies are needed in this regard. In light of all this information, the present study aims the using DCNN during the planning of implants which is a popular treatment option in dentistry practice.

In the literature, there are studies reported the AI systems' using in a variety of dentistry situations such as detecting dental caries [26–29], root fractures [30–32], root morphologies [33], jaw pathologies [34], periodontal bone damages [35–38], periapical lesions [21] and also determinating teeth and their numbering [19, 39]. Analyzes of these studies, which are the pioneers of DCNN applications in dentistry, were made on a wide variety of radiographic imageries such as periapical, panoramic, bitewing cephalometric, CT and CBCT. Nevertheless, it is seen that the number of studies based on CT and CBCT is limited [25]. Johari et al. found that the probabilistic neural network (PNN) method was successful in determining vertical root fractures in their study on CBCT images [31]. Hiraiwa et al., also reported that AI showed acceptable results in determining the extra roots of teeth in CBCT [33]. Also, Orhan et al. reported in their study on periapical lesions in CBCT images that the volume measurements calculated with the convolutional neural network (CNN) method are compatible with manual measurements and this situation are promising for the future [21].

Treatment planning is one of the most important steps of workflow in both medicine and dentistry. For the success in the treatment, the correct diagnosis should be made first, then the ideal treatment planning should be created for the patient. Treatment planning is a detailed organizational process; it depends on many factors such as the physician's knowledge experience [40]. In recent years, artificial intelligence systems have been used to support decision making processes in the diagnosis and treatment planning of physicians. The neural network machine learning system was used in various treatment plans such as radiation therapy and orthognathic surgery and promising results were obtained [40–42]. As it is known, radiographic imaging plays an important role in the planning of dental implants. It is recommended to examine the operation site with 3-dimensional imaging systems before the operation and to make detailed planning by performing a series of measurements under conditions permitted by anatomic variations [5]. Formations such as mandibular canal, sinuses and nasal fossa evaluated in the current study are the main anatomic variations that shape the implant planning. Kwak et al. recently reported successful results in determining the mandibular canal by the CNN method in CBCT images and stated that this may be an opportunity for future dental planning [18]. Similarly, Fukuda et al. evaluated the relationship between the 3rd mandibular molar tooth and the mandibular canal in their study on 600 panoramic radiographs [43]. Jaskari et. al have used the CNN method for mandibular canal segmentation in al CBCT images. They stated that AI systems give sensitive and reliable results in canal determination and these systems may be an important role in future implant planning [44]. The results of our study were similar to these studies; and its success percentage was 97.9% in the mandibular canal detection.

In the present study, sinus/fossa and missing teeth detection analysis were also performed, and it was observed that AI systems showed a success of 66.4% and 95.3%, respectively. It is seen that the number of studies regarding the detection of nasal fossa should be increased and the system should be improved. Because, as the success of the detection of anatomic structures in AI systems increases, the measurements made for implant planning will yield more successful results. To our best knowledge, there is no study in the literature for the determination of lost tooth/fossa and sinus, this is the first study on this subject. However, one study for determining sinus pathologies on panoramic images has been carried out and successful results of AI have been reported [45].

In this study, two separate measurements, bone thickness, and height were performed to evaluate the success of implant planning. The results of the study show that bone thickness measurements of AI should be improved using a deep learning system. We think this may be due to the measurement of the AI system with incorrect angles when evaluating bone thickness measurements. In the determination of nasal fossa in the maxilla and mandibular accessory canals in the mandibula, the system was not very sensitive; this situation caused some incompatibilities in the anterior region bone height measurements.

While the determination of the mandibular canal was successful, it is found that the bone height could not be determined correctly in these regions. We think that this may be because the system did not take into account the implant diameter and thickness. This deficiency may cause the system to bypass the canal from the buccal/lingual of the canal during measurement. As a result, the AI system can report measurements in this region higher than they should.

However, it is seen that the results of the AI system consistent with the manual measurements in the maxilla molar/premolar region, as well as in the mandible premolar region; these results offer hope for the usability of the system in implant planning.

## Conclusion

Consequently, using these systems in implant planning will both facilitate the work of physicians and will be a support mechanism in implantology practice. The success of the present study in the detection of sinus / mandibular canal and missing teeth and the measurements it offers in implant planning reinforces this possibility. There is a need for more extensive studies in which environmental anatomical formations are evaluated by AI for the development of CNN systems in implant planning.

## Data Availability

Not applicable.

## Abbreviations

AI: Artificial intelligence
CBCT: Cone-beam computed tomography
CT: Computed tomography
3D: Three-dimensional
CAD: Computer-aided diagnosis
DCNN: Deep convolutional neural network
TEM: Technical error measurement
PNN: Probabilistic neural network
CNN: Convolutional neural network
